# Needle stick and sharp injuries (NSSI) in health care workers in Ulaanbaatar

**DOI:** 10.1186/1753-6561-5-S6-P226

**Published:** 2011-06-29

**Authors:** G Narantuya, M Tsolmon

**Affiliations:** 1 Research and Surveillance of HAI, National Center for Communicable Disease, Ulaanbaatar, Mongolia

## Introduction / objectives

The study’s main objectives were to analyze the prevalence and assess the knowledge, attitude and practices (KAP) of health care workers regarding needle stick injuries in hospitals in Ulaanbaatar, Mongolia. In addition we also tried to determine the circumstances and type of injuries from needle sticks (such as from syringes, scalpels etc).

## Methods

It was a cross sectional survey conducted in two main hospitals in Ulaanbaatar. A total of 621 health care workers were recruited through a semi-structured questionnaire.

## Results

Health worker working 3 or more nights duties/week and more than 36 hours duty per week (p<0.05) were more prone to injuries.

The prevalence of NSSI in both the hospitals was 840 / 1000 HCW / year (84%) and majority of injuries occurred among nurses (p<0.00) besides laboratory assistants and housekeepers. A large amount of injuries occurred in injection room and in-patient departments. In majority of cases the common cause of injury was disposable syringe followed by needle on introven line and medication ampoule. Index finger was the most common site for injury. Most injuries occurred during recapping, opening of ampoule or vial and improper disposal of syringes.

Many (66%) had injury of moderate nature skin was punctured and some bleeding also occurred. Majority (80%) didn’t report injuries to hospital administration and neither seeks any treatment (75%) after injuries. Many consider needle re-sheathing the needle not important, which may lead to injuries to house keepers. We also found that majority had no training on NSSI and it was clear that incidence of injuries were less among trained health workers (p<0.00).

## Conclusion

To summarize the prevalence of NSSI among health care workers was very high.

## Disclosure of interest

None declared.

